# N-Glycosylation as a Tool to Study Antithrombin Secretion, Conformation, and Function

**DOI:** 10.3390/ijms22020516

**Published:** 2021-01-06

**Authors:** Sonia Águila, Rosina Noto, Ginés Luengo-Gil, Salvador Espín, Nataliya Bohdan, María Eugenia de la Morena-Barrio, Julia Peñas, Maria Carmen Rodenas, Vicente Vicente, Javier Corral, Mauro Manno, Irene Martínez-Martínez

**Affiliations:** 1 Servicio de Hematología y Oncología Médica, Hospital Universitario Morales Meseguer, Centro Regional de Hemodonación, Universidad de Murcia Campus Mare Nostrum, IMIB-Arrixaca, 30008 Murcia, Spain; sonia.aguila@um.es (S.Á.); gines.luengo@um.es (G.L.-G.); salvaalmudema@gmail.com (S.E.); nataliabhn@hotmail.es (N.B.); uge2985@hotmail.com (M.E.d.l.M.-B.); julia.penas@um.es (J.P.); mariacarmen.rodenas1@um.es (M.C.R.); vicente.vicente@carm.es (V.V.); javier.corral@carm.es (J.C.); 2 Institute of Biophysics (IBF), National Research Council of Italy (CNR), 153–90146 Palermo, Italy; rosina.noto@cnr.it (R.N.); mauro.manno@cnr.it (M.M.); 3 Grupo de investigación CB15/00055 del Centro de Investigación Biomédica en Red de Enfermedades Raras (CIBERER), Instituto de Salud Carlos III (ISCIII) Madrid, 28029 Madrid, Spain

**Keywords:** Glycosylation, folding, antithrombin, serpin, coagulation, thrombosis, bioengineering

## Abstract

N-linked glycosylation is a crucial post-translational modification involved in protein folding, function, and clearance. N-linked glycosylation is also used therapeutically to enhance the half-lives of many proteins. Antithrombin, a serpin with four potential N-glycosylation sites, plays a pivotal role in hemostasis, wherein its deficiency significantly increases thrombotic risk. In this study, we used the introduction of N-glycosylation sites as a tool to explore what effect this glycosylation has on the protein folding, secretion, and function of this key anticoagulant. To accomplish this task, we introduced an additional N-glycosylation sequence in each strand. Interestingly, all regions that likely fold rapidly or were surrounded by lysines were not glycosylated even though an N-glycosylation sequon was present. The new sequon in the strands of the A- and B-sheets reduced secretion, and the B-sheet was more sensitive to these changes. However, the mutations in the strands of the C-sheet allowed correct folding and secretion, which resulted in functional variants. Therefore, our study revealed crucial regions for antithrombin secretion and could potentially apply to all serpins. These results could also help us understand the functional effects of natural variants causing type-I deficiencies.

## 1. Introduction

Antithrombin is the most important natural anticoagulant, as its deficiency is associated with the highest risk of thrombosis [1]. Antithrombin deficiency is a rare congenital disease (1 in 3.000–5.000) [2], mainly caused by mutations in *SERPINC1*, the gene encoding this anticoagulant. Antithrombin deficiencies are classified as type I, when the mutation causes the absence of the protein in circulation, or type II, when the mutation allows for the presence of antithrombin variants in the plasma with null or impaired inhibitory activity [3]. Type I deficiency can be explained by mutations destabilizing the RNA or protein. Among the last group of defects, frameshifting, splicing, and nonsense mutations usually result in truncated proteins. Additionally, certain missense mutations can cause type I deficiencies through their effects on protein folding [4] since misfolded proteins can degrade in lysosomes or accumulate inside the endoplasmic reticulum [5]. As a member of serpins (serine protease inhibitors), antithrombin is characterized by great structural flexibility facilitating its efficient inhibitory capacity. However, this flexibility also favors abnormal folding into hyperstable conformations (latent or polymer), with a central sheet formed by six beta strands and a consequent loss of function [6,7]. Additionally, intracellular polymers are retained inside the endoplasmic reticulum, establishing aggregates that may cause toxicity (serpinopathy). In some serpins, secreted polymers have been detected [8]. In the case of antithrombin, disulfide-linked dimers have been observed in plasma from patients with missense mutations, thereby inducing intracellular polymerization [9].

Antithrombin has three beta-sheets (A-C), nine alpha-helices (A-I), and a reactive center loop. A feature that distinguishes antithrombin from other serpins is the presence of three disulfide bonds (using the numbering of the mature protein: Cys8-Cys128, Cys21-Cys95, and Cys247-Cys430). Additionally, the amino acid sequence presents 4 N-glycosylation sites (UniProtKB-P01008 (ANT3_HUMAN)). However, the Asn135 is not efficiently glycosylated, resulting in two detected glycoforms of antithrombin in plasma: Alpha, with 4 N-glycans, and beta, with 3 N-glycans [10]. N-glycosylation requires a consensus sequon Asn-X-Ser/Thr/Cys (X can be any amino acid except Pro) [11], and, in the case of antithrombin, the N-glycans incorporated are complex, biantennary, and feature terminal sialic acids. Typically, N-glycosylation takes place within flexible protein structures such as loops or helices, allowing interactions between the Asn in the consensus sequence and the oligosaccharide transferase complex (OST) [12,13]. Furthermore, it is well known that protein glycosylation occurs during folding [14]; therefore, we hypothesized that the folding of the strand results in an inaccessible consensus sequence for OST to transfer the oligosaccharide precursor to the Asn. Hence, the introduction of a new N-glycosylation site in each strand of this serpin could help elucidate the folding order of antithrombin. Moreover, the presence of new N-glycosylation in antithrombin might provide new clues related to the secretion, half-life, and function of this serpin, which could also be explored therapeutically.

## 2. Results

### 2.1. Expression of Recombinant Mutants

**Figure 1:**
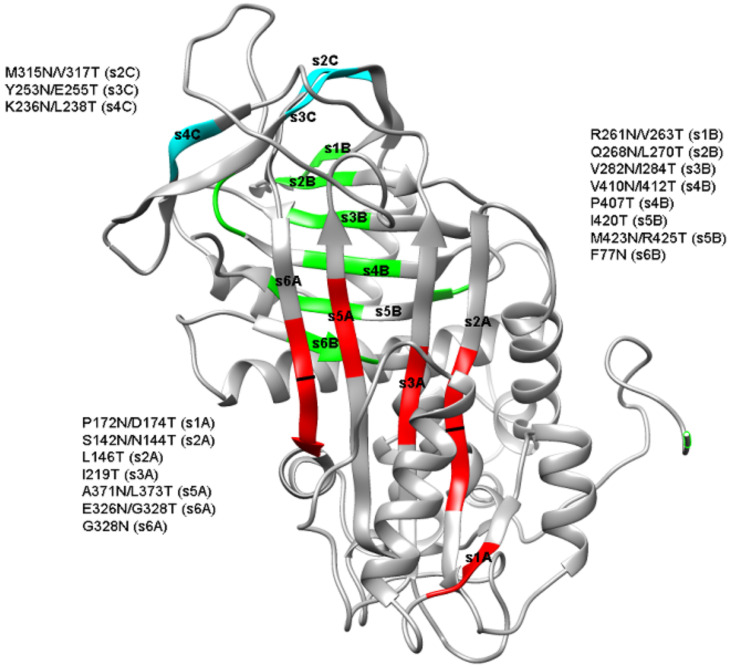
Structural representation of the mutations generated in the beta-strands of antithrombin. The consensus sequences generated at each strand are colored depending on the sheet to which they belong (red for the A-sheet, green for the B-sheet, and cyan for the C-sheet). Images were rendered with Pymol using Protein Data Bank (PDB): 1t1fA as a template.

We generated 18 different mutants, each one containing an additional N-glycosylation consensus sequence located in different strands of antithrombin. Figure 1 shows the locations of the mutations and the potential localizations of the additional N-glycans. 

**Figure 2:**
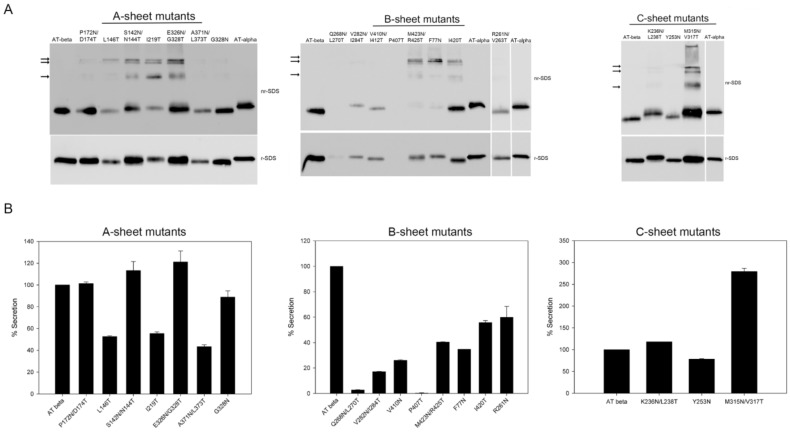
Secretion of mutant antithrombins. (**A**) SDS-PAGE under reducing and non-reducing conditions and Western blot of the medium after 48 h of transfection. (**B**) The rate of secretion of mutant antithrombins. The quantification of secretion was carried out by gel densitometry 48 h after transfection. Blots cropped from different gels are delineated with white space. Full-length gels are included in the Supplementary File.

We evaluated the incorporation of the extra glycan in all of the mutants by following their electrophoretic migration after secretion. As shown in Figure 2A, the mutations introduced within the A-sheet resulting in the addition of an extra-glycan were S142N/N114T, I219T, and E326N/G328T. Among the mutations introduced within the B-sheet, V282N/I284T, M423N/R425T, and F77N resulted in extra-glycosylation. Within the C-sheet, K236N/L238T and M315N/V317T mutants were extra-glycosylated. In all cases, the secreted mutants that were extra-glycosylated presented disulfide-linked polymerization, as shown under non-reducing conditions, except for V282N/I284T and K263N/L238T mutants.

We also estimated the secretion rate of each mutant after 24 h of transfection. Mutants within the A-sheet had similar secretion to the wild type protein, except for L146T, I219T, and A371N/L373T. All mutants within the B-sheet had impaired secretion, essentially null for Q268N/L270T and P407T mutants, as it was not possible to evaluate the extra glycan acquisition (Figure 2B). The secreted mutants within the C-sheet were not reduced, suggesting that the C-sheet is less sensitive than the A- or B-sheets to changes introducing new N-glycans or new mutations. This observation was consistent with the findings observed in patients with antithrombin deficiency carrying natural mutations. In our cohort of 350 unrelated cases with congenital antithrombin deficiency recruited during the last 20 years, missense mutations within the C-sheet did not dramatically alter protein secretion, with most having a pleiotropic effect on antithrombin. Interestingly, there was a mutant in the C-sheet (M315N/V317T) with the presence of an extra N-glycan whose secretion was ~2.7-fold higher than that of the wild type (Figure 2A,B). 

### 2.2. Thrombin and FXa Complex Formation

**Figure 3:**
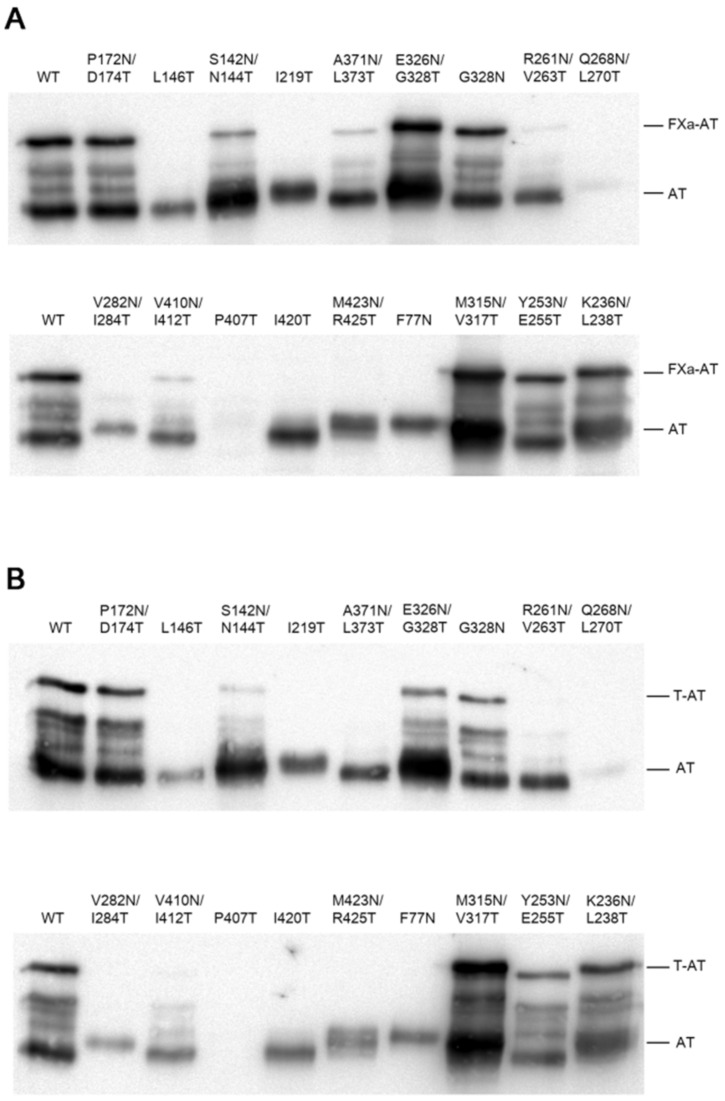
Inhibitory activity of antithrombin mutants. SDS-PAGE under the reduction and Western blot of the medium after 48 h of transfection and later incubation in the presence of low molecular weight heparin or unfractionated heparin with FXa (**A**) or thrombin (**B**). Full-length gels are included in the Supplementary File.

The inhibitory capacity of each mutant was also studied by evaluating the formation of antithrombin–thrombin or antithrombin–FXa complexes in the presence of heparin. We carried out this experiment using the 16 secreted mutants. Four of them (25%), I219T, R261N/V263T, I420T, and F77N, were not able to form complexes with the target proteases (Figure 3 and Table 1). However, most of the mutants that incorporated the extra N-glycan still retained their inhibitory activity. Interestingly, the variant A371N/L373T located within the strand s5A of the A-sheet, flanking the loop internalization following the complex formation, did not form a thrombin–antithrombin complex but was able to inhibit FXa (Figure 3). This result reflects the differences between thrombin and FXa inhibition by antithrombin during loop internalization, which was previously described [15,16,17]. Similar results were obtained when the inhibitory activity of the antithrombin variants was assayed in the absence of heparin (data not shown). 

### 2.3. Stability and Secondary Structure Determination

Latent antithrombin and polymers are two conformations characterized by the incapacity to inhibit the target proteases. They are also characterized by the presence of a sixth β-strand within the A-sheet via the insertion of the reactive center loop. Circular dichroisms (CD) were used to determine the content of secondary structural elements and record the transition from native to latent or polymeric conformations. We selected 3 mutants (L146T, F77N, and M315N/V317T) to compare the resulting spectra with those of the wild type protein. Each one of these mutants was representative of a mutant for each sheet of antithrombin and the different scenarios. L146T was not extra-glycosylated and did not show activity. F77N was poorly secreted but was extra glycosylated but showed no activity. M315N/V317T was both functional and extra-glycosylated. To address the structural features of each mutant, we performed an additional purification step via size exclusion chromatography. The purification profiles of L146T and F77N were significantly different from those of the wild type (Supplemental Figure S1), showing aggregates or oligomers that were removed for the CD spectra analysis. The CD spectra of isolated monomeric species showed very small differences confirming that all the mutants were correctly folded with the typical antithrombin secondary structural elements (Table 2).

**Figure 4:**
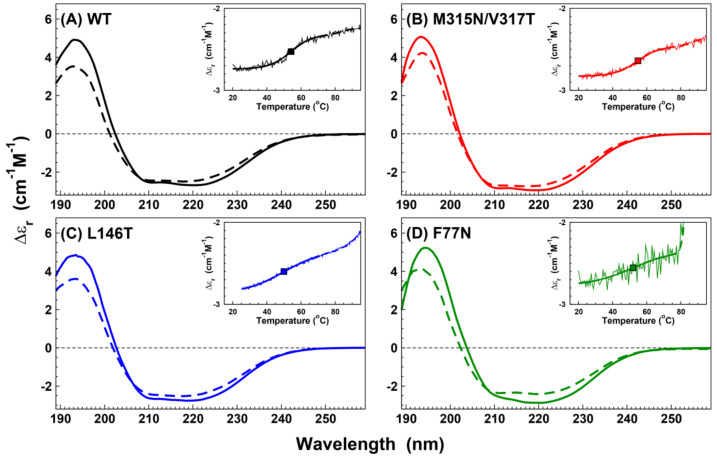
Secondary structures of the antithrombin mutants. Circular dichroisms (CD) spectra of wild type beta-antithrombin (S137A) (**A**) and mutants M315N/V317T (**B**), L146T (**C**), and F77N (**D**) for antithrombin monomers (solid curves) and polymers (dashed curves). Insets: CD signal at 221 nm on a thermal ramp (light curves); data are fitted here with sigmoidal curves (bold solid curves); the mid-point temperatures, shown as squares, are 54, 55, 50, and 52 °C for the wild type, M315N/V317T, L146T, and F77N, respectively; the onset of a second sigmoidal curve at higher temperatures indicates the onset of unfolding (dashed curves).

Changes in the secondary structure with temperature were measured. The transition observed in all mutants was likely due to latentization and/or polymerization processes, as in other serpins [18,19], while a sign of unfolding could be observable beyond 80 °C (insets of Figure 4A–D). However, in the case of F77N, and to a lesser extent for L146T, the unfolding sign was observed just below 80 °C. The samples were cooled down to 20 °C, and the CD spectra were recorded again. As shown in Figure 4A–D, the spectra after thermal stress exhibited a reduction in the signal around 220 nm; this feature highlights the typical pattern of polymeric serpins, as shown in analogous cases [20]. A more quantitative estimate was obtained by fitting the CD spectra using the web platform DichroWeb [21] with different algorithms and reference spectra (Table 2) [22]. While the unordered contribution was likely overestimated by the available reference sets, we noted that few differences were observable among the different mutants, which presented close secondary structures. Although it is important to note that the peak of F77N by gel filtration designated as a monomer was reasonably mixed with the tail of the oligomeric peak (Supplemental Figure S1), both F77N CD spectra were similar to the other variants. Thus, the spectrum after the thermal ramp was comparable to the “polymer” spectrum of the other variants, suggesting that the structure initially had a monomer-like conformation.

## 3. Discussion

Serpin polymerization has been a popular topic for many years [7,23,24,25,26,27,28,29,30,31]. Currently, there is still debate about the mechanism of polymerization. Moreover, it is not clear if such a mechanism may be different among serpins and if polymerization occurs through different mechanisms within the same serpin [32]. In the case of antithrombin, polymerization causes antithrombin deficiency and leads to increased thrombotic risk [9]. The characterization of antithrombin polymerization is very challenging. For example, proteins expressed in *Escherichia coli* result in the large production needed for these studies, but N-glycosylation is missing, thereby impacting antithrombin folding and secretion [33]. Interestingly, the presence of three disulfide bridges in the tertiary structure of antithrombin allowed this serpin to form disulfide linked dimers through monomeric disulfide bridges when expressed in eukaryotic cells. Theses bridges are easily detected using SDS gels without reducing agents. Indeed, these oligomers have also been described to allow polymerization in the plasma of patients with mutations [9]. In this study, we used N-glycosylation, a post-translational modification, to study the folding, secretion, and function of antithrombin, which may help us understand serpin misfolding. However, the creation of new N-glycosylation sequons in each strand of antithrombin had heterogeneous consequences, likely explainable not only by the presence of an additional N-glycan but also by the associated missense change(s) (Supplemental Figure S2). 

Our study provided intriguing results. First, although the mutations generated new N-glycosylation sequons, the glycosylation was not efficient in all cases (Table 1). Indeed, in eight of the sixteen secreted mutants, the consensus sequence was not glycosylated. This may be explained by two mechanisms: A) Other residues surrounding the N-glycosylation signal (N-X-S/T) modulated the efficiency of glycosylation. Thus, an aromatic residue preceding the asparagine increased the efficiency [34]. Recently, our group demonstrated that the presence of lysine residues close to the sequon impairs glycosylation efficiency (de la Morena Barrio et al., manuscript in preparation). Interestingly, in our present study, lysine residues were found in five out of the eight variants that were secreted and not glycosylated (Table 3), confirming the relevance of these electropositive residues in modulating the efficacy of N-glycosylation. The two non-glycosylated mutants without lysines at positions up to +4 or −4 from the asparagines did not have surrounding lysines in their 3D structures. B) Alternatively, the sequon may be present within a region that is immediately folded and does not allow the enzymatic action of the OST. Notably, the remaining two variants that were not glycosylated were located within the C-terminal hairpin (strands 4B and 5B). This is one of the first folded regions [35], supporting our hypothesis that the rapid folding of this region evades the action of the OST. 

Secondly, the introduction of new N-glycosylation sequences may have influenced the folding of the protein. Antithrombin is folded into a metastable native conformation, which may transform into a hyperstable latent or polymer conformation via different environmental factors, such as increased temperatures or pH modifications [36]. Indeed, like other serpins, even missense mutations may increase the rate of latent or polymer formation [37,38]. The identification of disulfide linked dimers in most variants with an additional N-glycan (particularly among those located within the A and B sheets) demonstrated that the presence of an extra N-glycan did not interfere with this polymerization process. Interestingly, this observation suggests that this additional post-translational modification may also affect the folding of antithrombin, thus favoring the transition to a hyperstable conformation. The gel filtration profiles showed significant differences for the L146T and F77N mutants compared to the wild type. The CD spectra clearly showed that the variants M315N/V317T, L146T, and F77N were able to polymerize. However, an early sign of unfolding was observed for the F77N variant, suggesting a different structural organization. Further studies will be required to clarify whether this effect might be caused by a missense change or by the new N-glycan in the case of the F77N variant. 

Third, we explored the functional consequences of additional N-glycans in this key anticoagulant serpin. Glycoforms lacking one of the N-glycans increased heparin affinity, but the inhibitory function was not affected [10]. Our study shows for first time that the presence of an extra glycan does not significantly interfere with antithrombin anticoagulant activity, except for I219T. This variant is located at strand 3A in a region that we have already shown to be critical for protease inhibition by affecting the rate of reactive loop insertion [15], possibly explaining the loss of functionality in this case. 

Finally, our study identified the mutant M315N/V317T displaying almost a 3-fold increased secretion compared to the WT. The single mutant already improved protein secretion (Supplemental Figure S2). Therefore, the potentially increased half-life of the hyperglycosylated protein remains to be determined, which might be a useful strategy to develop recombinant antithrombin with improved therapeutic usefulness. 

In conclusion, our study identified several locations in the A- and B-sheets of antithrombin as important for the proper folding of this protein. The introduction of N-glycosylation consensus sequences was used as a strategy to detect key strands for antithrombin folding by following the secretion and functionality of the protein. Such a strategy could be useful for studying the folding of other serpins or proteins. 

## 4. Materials and Methods

### 4.1. Recombinant Expression of Wild Type and Mutant Antithrombins

The mutations were constructed on the recombinant β-glycoform antithrombin (S137A) background to reduce glycosylation heterogeneity, as previously described [39]. Using the pCEP4-S137A/AT plasmid, recombinant antithrombin was only glycosylated at positions N96, N155, and N192. The numbering of the residues refers to the mature protein without the 32 amino acids of the signal peptide. Site-directed mutagenesis of the pCEP4-S137A/AT plasmid was performed using a Stratagene QuikChange Site-Directed Mutagenesis kit (Agilent Technologies, Santa Clara, CA, USA) and the appropriate primers for the mutations to obtain mutants with a consensus sequence for N-glycosylation (Asn-X-Ser/Thr). Single or double mutations were generated in each strand of the molecule to express the consensus sequence depending on the presence of one of the two required residues for the consensus sequence (Asn or Ser/Thr). Mutations were performed in non-conserved residues based on the multiple alignment of the amino acid sequences across different serpins and from different species [40] to avoid deleterious effects by those mutations. The antithrombin mutants did not contain any tag or other extra sequences. Human embryonic kidney cells expressing Epstein–Barr nuclear antigen 1 (HEK-EBNA) were grown to 60% confluence at 37 °C, 5% CO_2_, in DMEM with a GlutaMAX-I medium (Invitrogen, Barcelona, Spain) supplemented with 5% fetal bovine serum (Sigma–Aldrich, Madrid, Spain). We transfected 200 µg/mL of the wild type and mutant plasmids for 30 min in OptiMEM with LTX (Invitrogen, Barcelona, Spain), as suggested by the manufacturer. After 24 h, the cells were washed with PBS and moved into a CD-Chinese Hamster Ovary medium (Invitrogen, Barcelona, Spain) supplemented with 4 mM L-glutamine and 0.25 mg/mL Geneticin (Invitrogen, Barcelona, Spain). Cells were grown at 37 °C for 10 days only in the mutants that were purified from the culture medium. The medium was harvested and replaced by a fresh medium every 2 days.

### 4.2. Electrophoretic Analysis

Polyacrylamide gel electrophoresis under denaturing conditions was performed essentially as indicated elsewhere [41]. After separation, proteins were transblotted onto a polyvinylidene difluoride membrane. Antithrombin was then immunostained with rabbit anti-human antithrombin polyclonal antibody (Sigma–Aldrich, Madrid, Spain), followed by a donkey anti-rabbit IgG–horseradish peroxidase conjugate (GE Healthcare, Barcelona, Spain), with detection performed via an Enhanced Chemiluminescence (ECL) kit (Amersham Biosciences, Piscataway, NJ, USA). The secretion rate at 24 h after transfection relative to the wild type protein was evaluated by densitometry using the ImageJ software. 

### 4.3. Thrombin–Antithrombin and FXa-Antithrombin Complex Formation

The formation of complexes between antithrombin and its target proteases was evaluated by the incubation of a conditioned medium with 0.2 µM thrombin (Calbiochem, Millipore, Madrid, Spain) or 0.2 µM FXa (Enzyme Research Laboratories, South Bend, IN, USA) at 37 °C for 30 min. The reaction was carried out with and without the previous incubation of antithrombin with 0.6 µM heparin for 10 min. These samples were evaluated with SDS-PAGE as indicated above.

### 4.4. Protein Purification

Mutant recombinant proteins were purified from media harvests by heparin affinity chromatography on HiTrap Heparin columns (GE Healthcare, Barcelona, Spain) using an ÄKTA Purifier (GE Healthcare, Barcelona, Spain) in 100 mMTris-HCl and 10 mM citric acid in a gradient from 0.15 to 2M NaCl. Fractions with antithrombin were applied to a HiTrap Q column (GE Healthcare, Barcelona, Spain). Finally, the proteins were eluted in 3 different peaks and desalted over 5 mL of HiTrap Desalting columns (GE Healthcare, Barcelona, Spain) [42]. All protein samples were filtered with 0.2 µm syringe filters and separated by gel filtration (with a phenomena BioSep S3000 column) on an HPLC device, LC-2010 AT Prominence (Shimadzu, Kyoto, Japan), equipped with a UV-vis photodiode array detector. The monomer fraction was recovered from 9.5 to 11 mL for the wild type protein and from 9 to 10.5 mL for the other mutants, respectively, concentrated via centrifuge Amicon 10k filters, and used for CD measurements.

### 4.5. Circular Dichroism

Far-UV CD spectra were measured using a J-815 spectro polarimeter (Jasco, Tokyo, Japan) equipped with a Peltier-type temperature-control system. The spectra were acquired with an average of 3 scans (3 nm bandwidth, 16 s response, 5 nm min^−1^ scan rate) and baseline-corrected by subtracting the buffer spectrum. The mean residue differential extinction coefficient Δε_res_ in M^−1^ cm^−1^ units was calculated as in [20] from the observed ellipticity θ in degrees using the expression Δε_res_ = θ/(32.982 N_res_d c), where d is the path length in cm (d = 0.01 cm), N_res_ = 432 is the number of antithrombin residues, and c is the protein molar concentration—24.5, 23.0, 6.8, and 7.3 μM, for the wild type, M315/V317T, F77N, and L146T, respectively. Sample concentrations were accurately measured by absorption spectroscopy by assuming an extinction coefficient at 280 nm of 0.65 cm^−1^ (mg/mL)^−1^ = 37,700 cm^−1^ M^−1^ [43], and the samples were put on a 0.1-mm quartz cuvette for CD measurements. Changes in the secondary structure with temperature were measured by monitoring the CD signal at 221 nm over the course of a thermal ramp from 20 to 95 °C at a rate of 1 °C/min. The mid-point temperature (at about 55–60 °C) T_1/2_ was calculated via fitting with a sigmoidal curve. Secondary structural elements were assigned from the Protein Data Bank (PDB) structure using the STRIDE algorithm [44].

## Figures and Tables

**Table 1:** Mutations generating new N-glycosylation sites in antithrombin and the resulting effects. The consensus sequence was not introduced within strand 1C because it would have completely eliminated the strand. In the case of strand 4A, we did not introduce a consensus sequence as it folded as a strand only in the latent conformation. Each mutant only had one extra glycan. The numbering of residues refers to the mature sequence of antithrombin.

Mutants	Structure Location	Extra Glycan	Secretion Level	Anti FIIa Activity	Anti FXa Activity	Asn 3D Exposition
P172N/D174T	Strand 1A	NO	Normal	YES	YES	YES
L146T	Strand 2A	NO	Low	NO	NO	YES
S142N/N144T	Strand 2A	YES	Normal	YES	YES	NO
I219T	Strand 3A	YES	Low	NO	NO	NO
A371N/L373T	Strand 5A	NO	Low	NO	YES	NO
E326N/G328T	Strand 6A	YES	Normal	YES	YES	YES
G328N	Strand 6A	NO	Normal	YES	YES	YES
R261N/V263T	Strand 1B	NO	Low	NO	NO	YES
Q268N/L270T	Strand 2B	NO	Nil	NO	NO	YES
V282N/I284T	Strand 3B	YES	Very low	NO	NO	NO
V410N//I412T	Strand 4B	NO	Very low	NO	YES	NO
P407T	Beginning of strand 4B	NO	Nil	NO	NO	YES
I420T	Strand 5B	NO	Low	NO	NO	YES
M423N/R425T	Strand 5B	YES	Low	NO	NO	NO
F77N	Strand 6B	YES	Low	NO	NO	NO
M315N/V317T	Strand 2C	YES	Very high	YES	YES	YES
Y253N/E255T	Strand 3C	NO	Normal	YES	YES	YES
K236N/L238T	Strand 4C	YES	Normal	YES	YES	YES

**Table 2:** Secondary structural elements of mutant antithrombin. The percentage of residues involved in the different secondary structure classes for the beta-wild type (WT) and variant antithrombins M315N/V317T, L146T, and F77N. The results calculated for the WT PDB structure 1T1F are reported for comparison. (Here, unresolved residues in PDB are considered as coil elements).

Mutants	Alpha-Helix	Beta-Sheet	Turn	Coil
	%	%	%	%
WT	27 ± 3	25 ± 3	17 ±4	31 ± 3
M315N/V317T	29 ± 2	22 ± 2	17 ± 4	32 ± 1
L146T	29 ± 2	23 ± 2	17 ± 4	31 ± 2
F77N	30 ± 2	23 ± 2	17 ± 4	30 ± 3
pdb:1T1F	28	27	25	20

**Table 3:** Influence of surrounding Lysines on the efficacy of N-glycosylation in the mutations generated in this study. Asparagine and threonine/serine in the consensus sequence for N-glycosylation are displayed in red bold text. The presence of lysine at positions up to +4 or −4 from the asparagine is also indicated in bold and underlined. The mutations in which the results were unexpected regarding the presence or absence of surrounding lysines are displayed in gray.

Mutation	Amino Acid Sequence	Presence and Position of Lysines	Extra N-Glycan
P172N/D174T	AKLQNLTF**K**EN	YES; K +4	NO
L146T	LVSANRTFGDK	NO	NO
S142N/N144T	S**K**LVNATRLFG	YES; K−3	YES
I219T	LVLVNTTYFHG	NO	YES
A371N/L373T	AFH**K**NFTEVNE	YES; K−1	NO
E326N/G328T	RFRINDTFSLK	NO	YES
G328N	RIEDNFSL**K**EQ	YES; K + 4	NO
R261N/V263T	**K**FRYNRTAEGT	YES; K − 4	NO
Q268N/L270T	AEGTNVTELPF	NO	YES
V282N/I284T	DITMNLTLPKP	NO	YES
V410N/I412T	RPFLNFTREVP	NO	NO
P407T	TF**K**ANRTFLVF	YES; K − 2	NO
I420T	EVPLNTTIFMG	NO	NO
M423N/R425T	TIIFNGTVANP	NO	YES
F77N	NDNINLSPLSI	NO	YES
M315N/V317T	LEEMNLTVHMP	NO	YES
Y253N/E255T	ASMMNQTG**K**FR	YES; K + 4	NO
K236N/L238T	ENTRNETFYKA	NO	YES

## Data Availability

The data presented in this study are available in this article or in supplementary material.

## Supplementary Materials

The following are available online at https://www.mdpi.com/1422-0067/22/2/516/s1, Figure S1: Gel filtration profile of purified mutant antithrombins, Figure S2: Secretion of antithrombin simple mutants.

## Abbreviations

OST	Oligosaccharide transferase complex
CD	Circular dichroism
PDB	Protein Data Bank
