# Supplementary material for: N-Glycosylation as a Tool to Study Antithrombin Secretion, Conformation, and Function
Source: Int J Mol Sci. 2021 Jan 6;22(2):516. doi: 10.3390/ijms22020516 (PMC7825591; doi:10.3390/ijms22020516)
Supplement: Supplementary file 1 [file ijms-22-00516-s001.pdf]

N-glycosylation as a tool to study antithrombin folding

Sonia Águila[1], Rosina Noto[2], Ginés Luengo-Gil[1], Salvador Espín[1], Nataliya Bohdan[1], María Eugenia de la Morena-Barrio[1,3], Julia Peñas[1], Maria del Carmen Rodenas[1], Vicente Vicente[1,3], Javier Corral[1,3], Mauro Manno[2], Irene Martínez-Martínez[1,3*]

[1]Servicio de Hematología y Oncología Médica, Hospital Universitario Morales Meseguer, Centro Regional de Hemodonación, Universidad de Murcia, IMIB-Arrixaca, Murcia, Spain.

[2]Institute of Biophysics (IBF). National Research Council of Italy (CNR). Palermo, Italy.

[3]Grupo de investigación CB15/00055 del Centro de Investigación Biomédica en Red de Enfermedades Raras (CIBERER), Instituto de Salud Carlos III (ISCIII) Madrid, Spain.

Figure S1. Gel filtration profile of purified mutant antithrombins.

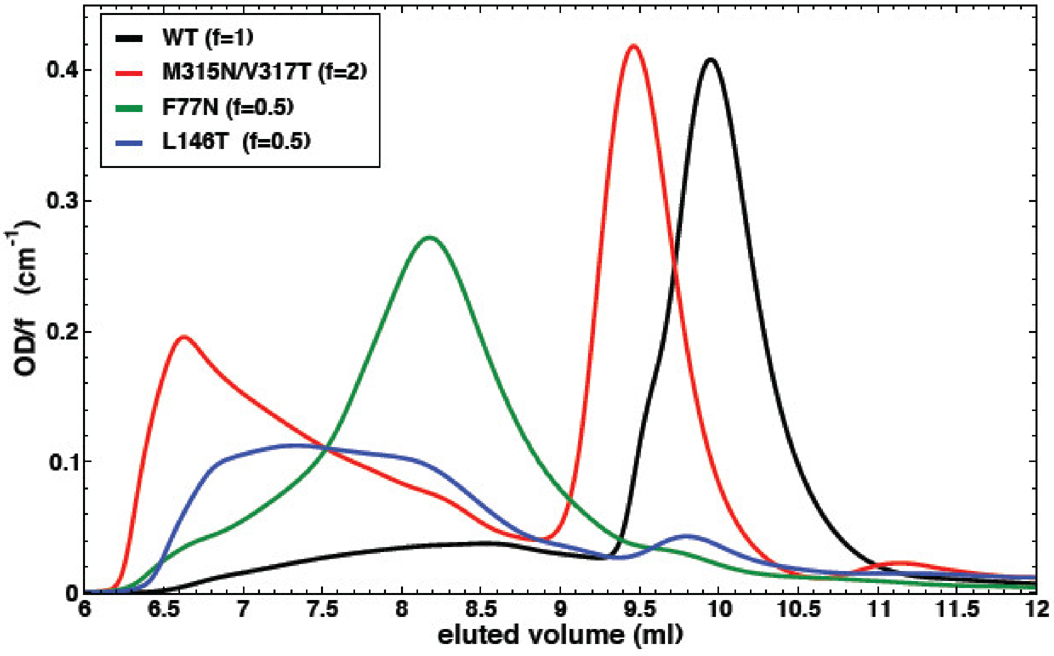

Figure S2. Secretion of antithrombin simple mutants. Secretion was evaluated in some of the double mutants carrying just one of the mutations. The M315N mutation was located within the C-sheet. R261N and V410N were located within the B-sheet, and the

A371N mutation was generated within the A-sheet. SDS-PAGE under reducing conditions and Western blot of the medium were performed after 48 hours of transfection.

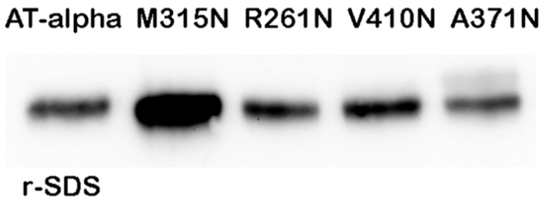

Supplemental information. Full-length blots of the gels contained in Figure 2.

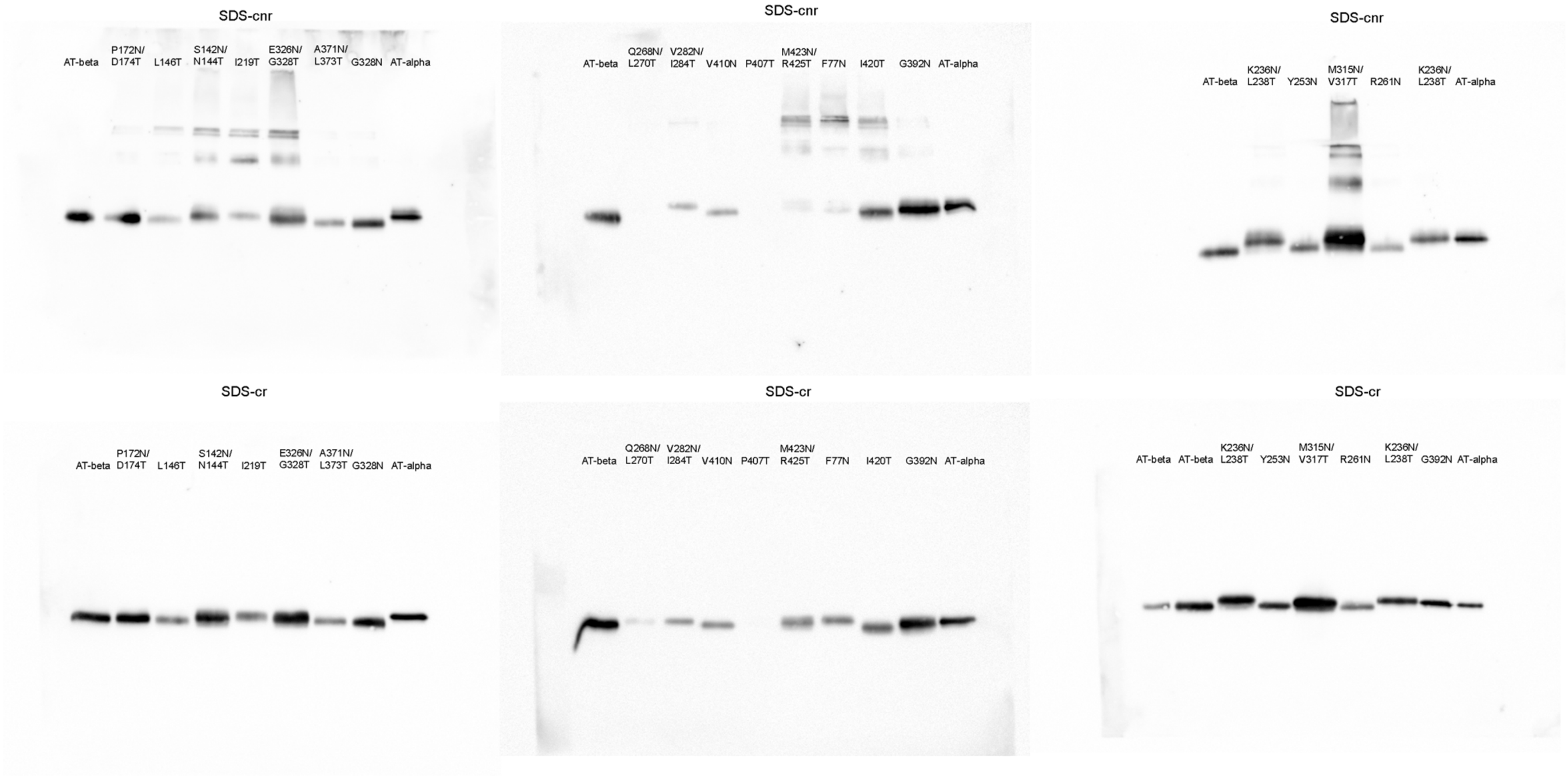

Supplemental information. Full-length blots of the gels contained in Figure 3.

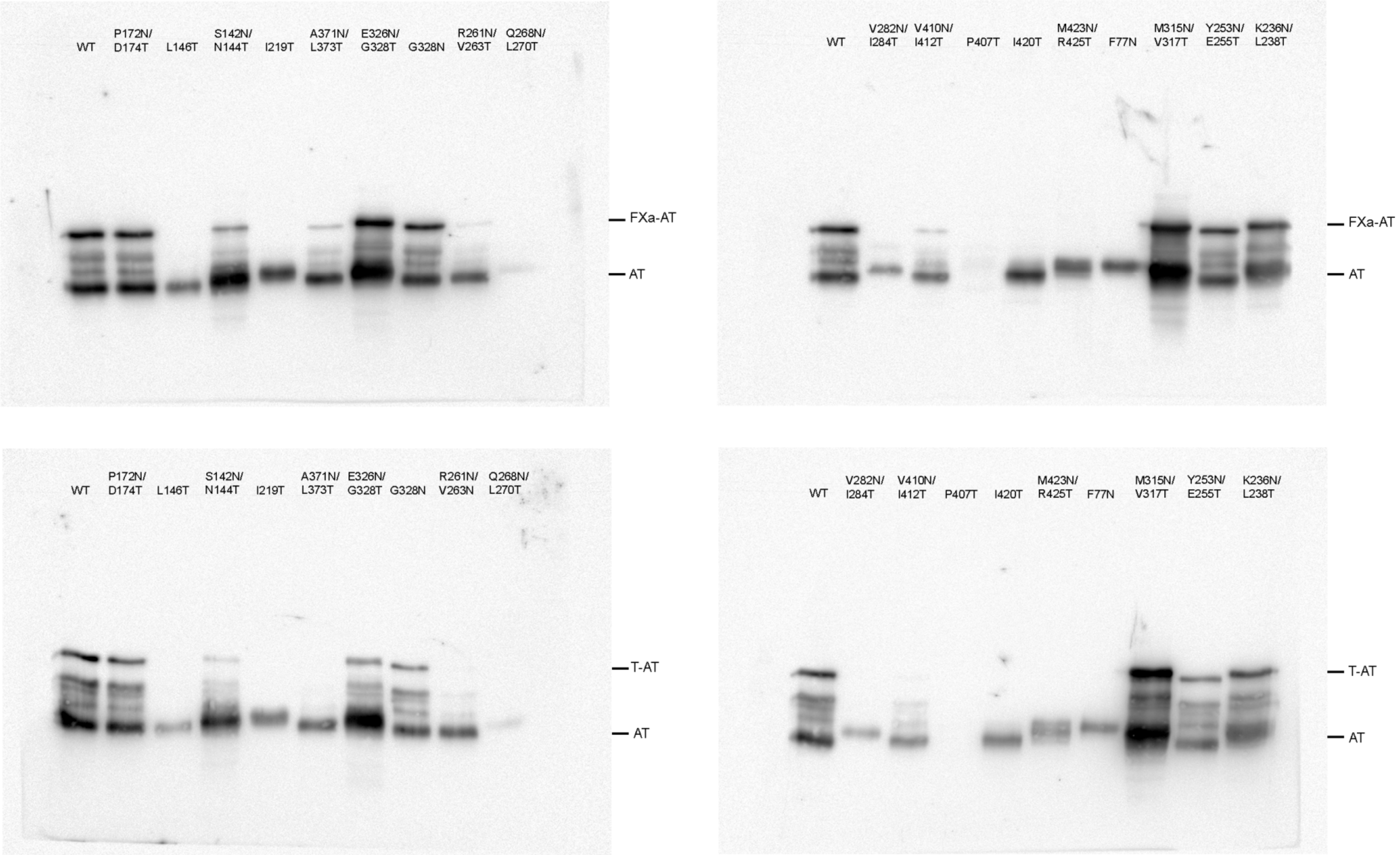
WT
P172N/ D174T
L146T
S142N/ N144T
I219T
A371N/ L373T
E326N/ G328T
G328N
R261N/ V263T
Q268N/ L270T
FXa-AT
AT
WT
V282N/ I284T
V410N/ I412T
P407T
I420T
M423N/ R425T
F77N
M315N/ V317T
Y253N/ E255T
K236N/ L238T
FXa-AT
AT
WT
P172N/ D174T
L146T
S142N/ N144T
I219T
A371N/ L373T
E326N/ G328T
G328N
R261N/ V263N
Q268N/ L270T
T-AT
AT
WT
V282N/ I284T
V410N/ I412T
P407T
I420T
M423N/ R425T
F77N
M315N/ V317T
Y253N/ E255T
K236N/ L238T
T-AT
AT
